# Quality of life among Jordanian patients on haemodialysis and their caregivers

**DOI:** 10.1186/1471-2458-12-S2-A14

**Published:** 2012-11-27

**Authors:** Emad A Shdaifat, Mohd Rizal Abdul Manaf

**Affiliations:** 1 Department of Community Health, Universiti Kebangsaan Malaysia Medical Centre, Jalan Yaacob Latiff, 56000 Kuala Lumpur, Malaysia

## Background

Quality of Life (QOL) is considered important outcome measure especially for chronic diseases such as chronic renal failure. The aims of the study were to measure the patients’ and caregivers’ QOL, and subsequently to compare QOL of patients and caregivers with general population.

## Methods

A cross-sectional study was performed on three Ministry of Health hospitals in Jordan from August to November 2010. Research and Development Corporation (RAND) 36-Item Health Survey 1.0 Arabic version was used to assess the QOL.

## Results

One hundred thirty-eight patients and forty-nine caregivers were involved in the study. Patients’ QOL score was less than their caregiver and both have poorer QOL compared to the general population. Patients’ and caregivers’ Physical Component Summary (PCS) were negatively correlated with their age (patients r=-0.38, P=0.001; caregivers r=-0.37, P=0.008). Moreover, caregivers’ Mental Component Summary (MCS) was negatively correlated with their age (r=-0.29, P=0.04). Single patients have higher PCS than married and widowed. Working patients have higher PCS than not working and retired. However, there was no correlation between patients’ and caregivers’ QOL (PCS P=0.32; MCS p=0.13).

## Conclusion

Patients’ and caregivers’ QOL were found to be significantly impaired compared to the general population. So, additional efforts need to be done to support patients and caregivers financially and to develop supportive groups in order to improve their QOL. Further interest should be given to older age and patients with family responsibilities, to help them cope with disease process and lifestyle changes.

